# Research on the influence of psychological fatigue of short track speed skaters on sports motivation

**DOI:** 10.3389/fpsyg.2026.1754610

**Published:** 2026-02-18

**Authors:** Guoxing Zhang, Feng Tai, Shihao Wang

**Affiliations:** Liaoning Normal University School of Physical Education, Dalian, China

**Keywords:** perceived social support, psychological fatigue, sense of career calling, short track speed skaters, sports motivation

## Abstract

**Objective:**

To clarify the mediating effects of perceived social support and sense of career calling on the relationship between psychological fatigue and sports motivation in short track speed skaters.

**Methods:**

Using a variety of research methods such as questionnaire survey and mathematical statistics, 817 short track speed skaters were selected as the research objects. The “Psychological Fatigue Scale,” “Sports Motivation Scale,” “Perceived Social Support Scale,” and “Sense of Career Calling Scale” were used for psychological measurement. SPSS 27.0 and PROCESS 4.2 macro program were used to test the mediating effect, chain mediating effect and Bootstrap analysis.

**Results:**

In the correlation analysis, psychological fatigue was significantly negatively correlated with perceived social support (*r* = −0.363, *p* < 0.01), sense of career calling (*r* = −0.387, *p* < 0.01), and sports motivation (*r* = −0.375, p < 0.01). Perceived social support was significantly positively correlated with sense of career calling (*r* = 0.494, *p* < 0.01) and sports motivation (*r* =0.505, p < 0.01). There was a significant positive correlation between sense of career calling and sports motivation (*r* = 0.520, *p* < 0.01).

**Conclusion:**

(1) Perceived social support and sense of career calling play a separate mediating role between psychological fatigue and sports motivation of short track speed skaters. (2) Perceived social support and sense of career calling play a chain mediating role between psychological fatigue and sports motivation of short track speed skaters. (3) Coaches should use strategies to improve perceived social support and sense of career calling of short track speed skaters, so as to improve the sports motivation of short track speed skaters and reduce their psychological fatigue.

## 1 Introduction

Psychological fatigue is a comprehensive phenomenon that short track speed skaters do not recover in time in the process of continuous consumption of physiological and psychological abilities in the face of various pressures, which leads to the decline of psychological function. This phenomenon will not only make short track speed skaters’ interpersonal relationship nervous, sports performance decline and motivation decline, but also endanger their mental health (Liu et al., 2023). In the process of competitive ability development, the cycle mode of “fatigue-adaptation-refatigue-readaptation” is the necessary adaptation process to improve the competitive ability of short track speed skaters. It points out that coaches should not only consider how to delay the occurrence of psychological fatigue, but also pay more attention to the regulation of the negative effects of psychological fatigue. In recent years, domestic existing literature research has pointed out that short track speed skaters generally have psychological fatigue in competitions (Zhao et al., 2022). Therefore, scientific management of the negative impact of psychological fatigue is of great value to improve the sports motivation of short track speed skaters. The decline of sports motivation is a typical feature after the occurrence of psychological fatigue. For a long time, the academic community has a strong interest in maintaining and enhancing sports motivation. Motivational integration theory believes that social environment factors and related psychological variables are the core determinants of enhancing sports motivation and guiding specific results (Wan et al., 2025), the integrated model of psychological fatigue explains that social support as an external buffer resource may reduce the negative impact of psychological fatigue on sports motivation (Gustafsson et al., 2011).

Social support refers to the material or spiritual support obtained by individuals from the outside world in a stressful environment, and gives individuals a sense of security, belonging and self-worth (Li and Li, 2024). The main effect and buffer effect model of social support explain that social support can enhance the development of individual mental health and buffer the negative impact of stressful events on individual body and mind. Perceived social support is an emotional experience that individuals are respected and understood in society, focusing on individual cognition and evaluation of support. In addition, sense of career calling is the core embodiment of the intrinsic value and long-term goals of short track speed skaters, and plays an important role between psychological fatigue and sports motivation. The sense of career calling of short track speed skaters not only affects their sports motivation and sports input, but also has an important impact on the fitness and satisfaction of their future careers (Yuan et al., 2022). Many studies have shown that when individuals have a sense of mission to a certain profession, they will highly recognize the profession and devote themselves to the activities of related professions. The sense of career calling will give individuals a sense of belonging to a specific profession and serve as an important carrier to realize self-concept (Birtch et al., 2021), and experience inner fun and self-realization in the profession. As an important psychological characteristic of short track speed skaters, the sense of career calling is also an important variable in this process. Accordingly, this study will study the relationship between “psychological fatigue, sports motivation, perceived social support and sense of career calling” of short track speed skaters and provide empirical evidence.

## 2 Research hypothesis

### 2.1 The relationship between psychological fatigue and sports motivation

Psychological fatigue refers to the state of exhaustion caused by long-term psychological activities or emotional stress, which is manifested as inattention, decreased decision-making ability and weakened motivation. Sports motivation refers to the internal or external factors that drive individuals to participate in sports, involving the combined effects of individual needs, goals and values. The improvement of sports motivation can encourage short track speed skaters to have better performance in training and competition (Shen et al., 2025). According to the dual tendency of approach and avoidance of sports motivation, sports motivation can be divided into two types: participation tendency motivation and avoidance tendency motivation. Existing research shows that one of the performances of short track speed skaters after psychological fatigue is that the level of sports motivation is significantly reduced, accompanied by a subjective level of escape intention (Tongtong and Erlin, 2018). The emergence of psychological fatigue leads to the weakening of the participation tendency motivation of short track speed skaters, and the avoidance tendency motivation is correspondingly enhanced. The empirical research results of short track speed skaters provide supporting evidence for this correlation (Guo, 2015). In addition, psychological fatigue is one of the important factors affecting the sports motivation of short track speed skaters, and it is also an important inducement affecting the mental health, training quality and competitive performance of short track speed skaters.

In view of this, this study proposes the hypothesis H1: psychological fatigue negatively affects the sports motivation of short track speed skaters.

### 2.2 The mediating effect of perceived social support between psychological fatigue and sports motivation

Perceived social support is the core concept of psychology and sociology, it is the individual’s subjective feeling and evaluation of the degree of support from the outside world (Zimet et al., 1988). The core is “subjective belief” rather than objective facts. Even if the actual support exists, if the individual does not understand it, it cannot play a role. Perceived social support and actual social support are relative concepts, both of which are important components of the structural elements of social support. The actual social support is the actual support obtained by the individual, and it is the actual help behavior provided by the surrounding people when the individual is under pressure. Perceived social support is an individual’s expectation and evaluation of social support, it is a belief in the possible social support (Sarason et al., 1990), which is manifested as the emotional experience of being supported, understood and respected in society (Sarason et al., 1991).

Perceived social support as a positive psychological resource can not only relieve stress and burnout, but also help self-regulate negative emotions (Fang et al., 2021). The buffer model of social support points out that perceived social support can provide important psychological resources and supplement the lost psychological energy. Perceived social support refers to the subjective attention and help obtained from social relations. It is characterized by emotional support, instrumental support and information support, which can come from multiple sources such as friends or family (Scardera et al., 2020). The perceived social support of short track speed skaters is the subjective judgment of short track speed skaters to help people from teammates, coaches and other groups when they need it. Perceived social support has better predictive ability than actual social support, and is often used as an alternative observation index for social support research. The negative emotions of short track speed skaters due to psychological fatigue will affect their daily training, which will induce interpersonal tension, and the change of interpersonal status will further affect the level of perceived social support of short track speed skaters. Previous studies have pointed out that the positive interaction between short track speed skaters and coaches will directly affect the subjective judgment of short track speed skaters on the accessibility of coaches support (Miao et al., 2022), so that short track speed skaters think that they can get help from coaches when they need help, and improve their perception of social support. At the theoretical level, the intermediary model of behavioral motivation theory and overall self-worth provides a mechanism explanation for understanding the relationship between perceived social support and sports motivation. At the same time, relevant research in the field of sports believes that external support can enhance the self-worth identity of short track speed skaters, and ultimately affect the intensity of sports motivation, that is, the physical exercise of short track speed skaters is not only affected by internal motivation, but also by external factors such as social support (Liao et al., 2025). Based on the above empirical and theoretical basis, psychological fatigue may indirectly affect the sports motivation of short track speed skaters by changing the level of perceived social support.

In view of this, this study proposes Hypothesis H2: Perceived social support plays an intermediary role between psychological fatigue and sports motivation.

### 2.3 The mediating role of sense of career calling between psychological fatigue and sports motivation

In addition to perceived social support, sense of career calling may also be another mediating variable in the relationship between “psychological fatigue” and “sports motivation.” Sense of career calling has attracted much attention in professional psychology and organizational behavior research at home and abroad (Duffy and Dik, 2013). It advocates that people regard career as the purpose and value of life, and people with higher sense of career calling pay more attention to the role of career in self-realization and the meaning of life, and are proud of their career, so as to improve their mental health (Hui et al., 2025). Specifically, the sense of career calling refers to the individual’s strong enthusiasm and interest in the profession (Hall and Chandler, 2005). It is a highly personalized intrinsic psychological motivation that is conducive to the completion of professional missions (Dik and Shimizu, 2018), which goes beyond simple livelihood needs or professional achievements. Scholars generally regard the sense of career calling as a sense of mission beyond the self, intended to achieve their own roles in a way that connects the career with the purpose and meaning of life, and takes altruistic values and goals as the primary motivation. The sense of career calling is a calling beyond the self to complete a specific life role in a way that shows or acquires a sense of purpose and is driven by the other as the main source of motivation (Erum et al., 2021). In addition, the sense of career calling is derived from the inner love of something, is an internal motivation, is a highly automated and self-determinative internal motivation, has a driving function that transcends the influence of other external factors, and can improve the individual’s psychological resilience (Zhong et al., 2025). Social identity theory holds that individuals, with the support of the outside world, carry out social classification according to certain standards, obtain group identity and get self-satisfaction, realize the support brought by the group, and then have a sense of mission and responsibility for the profession, so they choose to remain in the group. If the individual does not identify with the collective, he or she cannot perceive the support brought by the group and cannot feel the sense of mission, then the individual will adopt the strategy of separating from the group to change the status quo. When short track speed skaters suffer from psychological fatigue caused by training, competition and other aspects for a long time, their sports motivation may be weakened. However, a higher sense of career calling can effectively resist this effect. By strengthening short track speed skaters’ deep understanding of the significance of their sports career and their awareness of team or social responsibility, an important transformation path between psychological fatigue and sports motivation is constructed. The sense of career calling provides sufficient feedback to reduce the potential negative impact of psychological fatigue on sports motivation (Holgado et al., 2023), and it counteracts the burnout and goal drift tendency brought by psychological fatigue, and transforms external pressure or internal consumption into the internal driving force to enhance sports motivation, thus forming a positive intermediary role between the two.

In view of this, this study proposes Hypothesis H3: Sense of career calling plays a mediating role between psychological fatigue and sports motivation.

### 2.4 Comprehension of the chain mediating role of psychological fatigue and sense of career calling between psychological fatigue and sports motivation

The chain mediating effect of perceived social support and sense of career calling between psychological fatigue and sports motivation refers to the fact that when short track speed skaters experience psychological fatigue, they can indirectly affect their dynamic mechanism of sports participation through perceived external assistance and internal professional identity. The theory of social support points out that the stronger the social support network that individuals have, the more they can cope with various challenges from the environment. Social support is the individual’s perception of the external support that they want or can get, and it is a factor that promotes individual development in the social environment. Specifically, the chain mediation model reveals how social support first enhances the individual’s sense of career calling, and then buffers the negative impact of psychological fatigue, and ultimately improves the level of sports motivation of short track speed skaters. The sense of career calling affects the individual’s awareness of the profession. According to the social cognitive career theory, the sense of career calling and outcome expectation can shape the individual’s professional interest and enhance the professional identity (Tang et al., 2022). People with a sense of career calling will be loyal to their professional roles, become excellent members in this field and realize social value. Therefore, only by establishing a good sense of career calling can individuals truly feel the satisfaction and sense of value brought by the profession (Zhang et al., 2016). In empirical research, short track speed skaters’ high-level perceived social support from teammates or coaches has been proved to significantly strengthen the short track speed skaters’s sense of career calling to engage in sports, from reducing psychological fatigue symptoms to stronger sports motivation. This mechanism not only explains how psychological resources are mobilized through multi-level mediation, but also provides a theoretical basis for intervention strategies, emphasizing the importance of strengthening social networks and professional identity in training or competition environments to maintain motivation.

In view of this, this study proposes Hypothesis H4: perceived social support and sense of career calling have a chain mediating effect between psychological fatigue and sports motivation of short track speed skaters.

**Figure 1:**
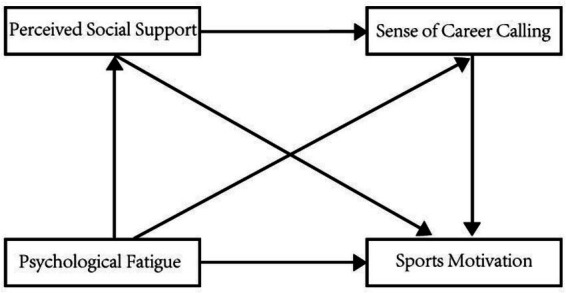
Research hypothesis model.

To sum up, the occurrence of psychological fatigue will reduce the sports motivation of short track speed skaters. As the key intermediary elements, perceived social support and sense of career calling may play an intermediary role and chain intermediary role in the influence of psychological fatigue on sports motivation. In order to test the relationship between psychological fatigue, sports motivation, perceived social support, and sense of career calling, this study proposes Hypothesis H1, Hypothesis H2, Hypothesis H3, and Hypothesis H4, and constructs a research hypothesis model, as shown in Figure 1.

## 3 Research objects and methods

### 3.1 Research object

Due to the particularity of the four variables of psychological fatigue, sports motivation, perceived social support and sense of career calling in the study, short track speed skaters aged 18 and over were selected for research. This study combines stratified sampling and cluster sampling. With the consent of all coaches and short track speed skaters, from September 1, 2025 to October 20, 2025, short track speed skaters were organized by coaches to fill out questionnaires online. A total of 823 questionnaires were distributed and screened after recovery. The final effective questionnaire was 817, including 611 males and 206 females. There are 465, 220, and 132 short track speed skaters aged 18–20, 21–23, 24 and above, respectively.

### 3.2 Research methods

In this study, mature questionnaires were selected, and only on the basis of the original questionnaire, individual questions were moderately adjusted according to the actual situation. Specifically, this study only adjusts the nouns of some questions in the questionnaire to a small extent, without changing the original meaning of the questions, so as to ensure that the revision will not affect the original reliability and validity of the questionnaire, and will not affect the reliability and validity of the questionnaire.

#### 3.2.1 Questionnaire survey method

The questionnaire is composed of short track speed skaters’ personal background information table, “Psychological Fatigue Scale,” “Sports Motivation Scale,” “Perceived Social Support Scale,” and “Sense of Career Calling Scale.”

##### 3.2.1.1 Personal background information table of short track speed skaters

The “personal background information table” of short track speed skaters includes information on the gender short track speed skaters.

##### 3.2.1.2 Psychological fatigue scale

The “psychological fatigue scale “compiled by Raedeke et al. and revised by Zhang and Mao (2010) was used for measurement. The scale includes three sub-dimensions: reduced sense of achievement, emotional or physical exhaustion, and negative evaluation of exercise, with a total of 15 items. Likert 5-level scoring method was used, 1 = never; 2 = very little; 3 = sometimes: 4 = often; 5 = always. The score of each question is the value of the answer selected for the question; reverse scoring questions, converted score = 6-the original score of the problem.

The Cronbach’s α of “Psychological Fatigue Scale” in this study was 0.856, and the Cronbach’s α of the three sub-dimensions was 0.912–0.931.

##### 3.2.1.3 Sports motivation scale

Tension was measured using “Sports Motivation Scale” developed by Zhang and Mao (2010). The scale includes two sub-dimensions, such as participation tendency and avoidance tendency, with a total of 6 items. Likert 5-level scoring method was used, 0 = completely disagree, 1 = disagree, 2 = uncertain, 3 = disagree, 4 = completely agree.

The Cronbach’s α of the “Sports Motivation Scale” in this study was 0.812, and the Cronbach’s α of the two sub-dimensions was 0.825 and 0.833, respectively.

##### 3.2.1.4 Perceived social support scale

The “Perceived Social Support Scale” in the field of sports compiled by Freeman et al. and revised by Qiu (2015) was used for measurement. The scale includes four sub-dimensions: emotional support, respect support, information support and tangible support, with a total of 16 items. A 7-point scale was used, 1 = disagree completely, 2 = disagree less, 3 = disagree slightly, 4 = neutral, 5 = agree slightly, 6 = agree, 7 = agree completely.

The Cronbach’s α of the “Perceived Social Support Scale” in this study was 0.892, and the Cronbach’s α of the four subdimensions was 0.804–0.842.

##### 3.2.1.5 Sense of career calling scale

The “Sense of Career Calling Scale” (compiled by Zhang Chunyu) included in the book “Handbook of Common Psychological Assessment Scales” (3rd edition) by Xiaoyang and other scholars was used for measurement (Xiaoyang et al., 2023). The scale includes three sub-dimensions: guiding force, meaning and value, and altruistic contribution, with a total of 11 topics. First, the guiding force, emphasizing that the mission is guided by a certain force to guide the individual to approach or accept a career; second, meaning and value, that is, emphasizing the sense of professional mission to link career with the meaning of life, the purpose of life, and the value of life; third, altruistic contribution, that is, the sense of professional mission contains a tendency to be altruistic, to want to help others, or to have a good impact on others. The guiding force includes four topics; meaning and value includes three topics; the altruistic contribution includes four topics. Using the Likert 5-level scoring method, 1 = completely inconsistent, 2 = relatively inconsistent, 3 = intermediate, 4 = relatively consistent, 5 = completely consistent.

The Cronbach’s α of the “Sense of Career Calling Scale” in this study was 0.879, and the Cronbach’s α of the three sub-dimensions was 0.824–0.874.

#### 3.2.2 Mathematical statistics

The questionnaire collected online was collated into EXCEL table data, and then imported into SPSS 27.0 statistical software to encode and name the variables, and descriptive analysis and correlation analysis of relevant variables were carried out. At the same time, reliability analysis and confirmatory factor analysis were used to test the reliability and validity of the scale. Pearson correlation analysis was used to detect the correlation between variables. The linear regression analysis and the PROCESS 4.2 macro program developed by Hays were used to test the significance of the mediating effect. The non-parametric percentile Bootstrap (5000) method was used to test the significance level of the mediating effect.

The specific settings when using the Process plug-in to analyze the indirect effects of variables are as follows: Set the model as Model (6), X = psychological fatigue, M1 = perceived social support, M2 = sense of career calling, Y = sports motivation.

## 4 Research results and analysis

### 4.1 Common method bias test

Before the questionnaire was issued and collected, the testers were trained professionally, tested according to the standardized process, and the common method variation was controlled by anonymous and reverse scoring of some items. However, because the research data were all from the subjective questionnaire survey, there may still be common method deviation. Therefore, the Harman single factor test method was used to test the common method deviation of the relevant variables.

Through the unrotational exploratory factor analysis, the results showed that there were 12 factors with eigenvalues greater than 1, and the variation explained by the first common factor was 23.629% which was less than the critical standard of less than 40% proposed by Podsakoff et al. (2003).

### 4.2 Difference test of different groups

In order to explore whether there are gender differences in short track speed skaters’ psychological fatigue, perceived social support, sense of career calling and sports motivation, the independent sample “*t*” test was used for analysis, as shown in Table 1. There is no significant difference between male short track speed skaters and female short track speed skaters in psychological fatigue (*p* > 0.05), perceived social support (*p* > 0.05), sense of career calling (*p* > 0.05), and sports motivation (*p* > 0.05).

**Table 1:** Gender differences between different variables.

Variant	Gender	Quantity	*M*	SD	*F*	*P*
Psychological fatigue	Male	611	3.23852	0.681801	2.174	0.155
	Female	206	3.31521	0.631577		
Perceived social support	Male	611	4.98670	0.810157	0.652	0.923
	Female	206	4.98058	0.727667		
Sense of career calling	Male	611	3.82443	0.626203	0.272	0.512
	Female	206	3.79126	0.634077		
Sports motivation	Male	611	2.84561	0.819938	0.496	0.439
	Female	206	2.79531	0.763166		

### 4.3 Descriptive statistics and correlation analysis between variables

Baron and Kenny (1986) believe that the prerequisite for the analysis of mediating effects is to determine the correlation between independent variables, dependent variables and mediating variables. Therefore, the study conducted a correlation analysis of psychological fatigue, sports motivation, perceived social support, and sense of career calling. The results showed that among the research variables, the skewness score was −1.252 to −0.222, and the kurtosis score was −0.431 to 2.558. The research shows that when the absolute value of skewness is less than 3 and the absolute value of kurtosis is less than 10, the sample can be considered to obey the normal distribution, and the skewness and kurtosis of the variable data meet the requirements. Therefore, the data basically conforms to the normal distribution and can be used for subsequent analysis.

In the correlation analysis, psychological fatigue was significantly negatively correlated with perceived social support (*r* = −0.363, *p* < 0.01), sense of career calling (*r* = −0.387, *p* < 0.01), and sports motivation (*r* = −0.375, *p* < 0.01). Perceived social support was significantly positively correlated with sense of career calling (*r* = 0.494, *p* < 0.01) and sports motivation (*r* = 0.505, *p* < 0.01). There was a significant positive correlation between sense of career calling and sports motivation (*r* = 0.520, *p* < 0.01), as shown in Table 2. The above correlation analysis results lay a good foundation for in-depth understanding of the internal relationship between variables.

**Table 2:** Descriptive statistics and correlation analysis between variables.

Variables	*M*	SD	Psychological fatigue	Perceived social support	Sense of career calling	Sports motivation
Psychological fatigue	3.25785	0.669942	1			
Perceived social support	4.98516	0.789739	−0.363^**^	1		
Sense of career calling	3.81607	0.627973	−0.387^**^	0.494^**^	1	
Sports motivation	2.83293	0.805837	−0.375^**^	0.505^**^	0.520^**^	

^**^At the 0.01 level (two-tailed), the correlation is significant.

### 4.4 Test of chain mediating effect of perceived social support and sense of career calling

There is a significant correlation between psychological fatigue, sports motivation, perceived social support and sense of career calling, which meets the statistical requirements for further analysis of the mediating effect of perceived social support and sense of career calling. The PROCESS 4.2 macro program in SPSS 27.0 was used to test the mediating effect, and the demographic variables (gender and age) were controlled. The Bootstrap sampling amount of 5,000 times was selected to test the chain mediating effect of perceived social support and professional mission in the 95% confidence interval.

Firstly, multiple regression analysis showed that psychological fatigue had a significant negative predictive effect on sports motivation (β = −0.374, *p* < 0.001). After psychological fatigue, perceived social support and sense of career calling were included in the regression equation, the negative predictive effect of psychological fatigue on sports motivation was still significant (β = −0.144, *p* < 0.001). Psychological fatigue had a significant negative predictive effect on perceived social support (β = −0.361, *p* < 0.001), and psychological fatigue had a significant negative predictive effect on sense of career calling (β = −0.238, *p* < 0.001). Perceived social support had a significant positive predictive effect on sense of career calling (β = 0.408, *p* < 0.001), and perceived social support had a significant positive predictive effect on sports motivation (β = 0.296, *p* < 0.001). The sense of career calling had a significant positive predictive effect on sports motivation (β = 0.319, *p* < 0.001). As shown in Table 3.

**Table 3:** The regression analysis of perceived social support and sense of career calling between psychological fatigue and sports motivation.

The regression equation		Overall fitting index			The significance of regression coefficient		
Outcome variables	Predictive variables	*R*	*R* ^2^	*F*	β	SE	*t*
Sports motivation	Gender	0.375	0.140	44.267^***^	−0.008	0.060	−0.261
	Age				−0.003	0.035	−0.089
	Psychological fatigue				−0.374	0.039	−11.481^***^
Perceived social support	Gender	0.366	0.134	41.870^***^	0.015	0.059	0.463
	Age				−0.047	0.034	−1.429
	Psychological fatigue				−0.361	0.039	−11.050^***^
Sense of career calling	Gender	0.542	0.294	84.615^***^	−0.010	0.043	−0.327
	Age				−0.006	0.025	−0.189
	Psychological fatigue				−0.238	0.030	−7.525^***^
	Perceived social support				0.408	0.025	12.866^***^
Sports motivation	Gender	0.608	0.369	94.933^***^	−0.012	0.052	−0.424
	Age				0.019	0.030	0.672
	Psychological fatigue				−0.144	0.037	−4.651^***^
	Perceived social support				0.296	0.034	8.992^***^
	Sense of career calling				0.319	0.043	9.610^***^

^*^p < 0.05, **p < 0.01, ***p < 0.001.

Secondly, the mediating effect test. Continue to use the Model (6) in the PROCESS 4.2 macro program to test the significance of the mediating effect of perceived social support and sense of career calling. The bias-corrected nonparametric percentage Bootstrap test was used to repeatedly sample 5,000 times and calculate the 95% confidence interval (CI). If 95% CI does not contain 0, it means that the mediating effect is significant. As shown in Table 4. Perceived social support and sense of career calling play a mediating role between psychological fatigue and sports motivation, the mediating effect value is −0.277, and its 95% confidence interval is [−0.342, −0.215]. The confidence interval does not include 0, indicating that the mediating effect is significant, accounting for 61.42% of the total effect of psychological fatigue on sports motivation (−0.451). The mediating effect includes three indirect effect paths: (1) The indirect effect 1 (−0.128) produced by the way of “psychological fatigue—perceived social support—sports motivation.” and the 95% confidence interval does not contain 0, indicating that the indirect effect of the mediating variable is significant, accounting for 28.38% of the total effect of psychological fatigue on sports motivation (−0.451); (2) The indirect effect 2 (−0.092) through the path of “psychological fatigue—sense of career calling—sports motivation,” 95% of the confidence interval does not contain 0, indicating that the indirect effect of the intermediary variable is significant, accounting for 20.40% of the total effect of psychological fatigue on sports motivation (−0.451); (3) The indirect effect 3 (−0.057) through the path of “psychological fatigue—perceived social support—sense of career calling—sports motivation,” 95% of the confidence interval does not contain 0, indicating that the indirect effect of the intermediary variable is significant, accounting for 12.64% of the total effect of psychological fatigue on sports motivation (−0.451).

**Table 4:** Analysis of the mediating effect of perceived social support and sense of career calling in the influence of psychological fatigue on sports motivation.

Effect situation	Effect value	Boot standard error	Boot CI lower limit	Boot CI upper limit	Relative mediating effect
Gross effect	−0.451	0.039	−0.527	−0.374	
Direct effect	−0.174	0.037	−0.247	−0.101	38.58%
Total indirect effect	−0.277	0.032	−0.342	−0.215	61.42%
Indirect effect 1 (Ind1): Psychological fatigue-perceived social support-sports motivation	−0.128	0.021	−0.173	−0.090	28.38%
Indirect effect 2 (Ind2): Psychological fatigue-sense of career calling-sports motivation	−0.092	0.016	−0.125	−0.063	20.40%
Indirect effect 3 (Ind3): Psychological fatigue-perceived social support-sense of career calling-sports motivation	−0.057	0.012	−0.082	−0.036	12.64%

## 5 Discussion

By introducing two variables, such as perceived social support and sense of career calling, this study constructs and verifies the chain mediating model of the relationship between psychological fatigue and sports motivation of short track speed skaters. This paper focuses on the chain mediating role of perceived social support and sense of career calling between psychological fatigue and sports motivation, which is helpful to clarify the influence mechanism of psychological fatigue on sports motivation, and is an active exploration to improve sports motivation. The total effect value of psychological fatigue on sports motivation was −0.451, the direct effect value was −0.174, and the total indirect effect value was −0.277. Perceived social support and sense of career calling, as well as the mediating effects of the two, reached a statistically significant level. The results show that the chain mediation model of the research hypothesis is established, and the four hypotheses are verified. From a theoretical point of view, it enriches the research on the influence mechanism of sports motivation and deepens the research on psychological fatigue. From a practical point of view, it provides a reference for reducing psychological fatigue, enhancing sports motivation, enhancing perceived social support, and enhancing the sense of career calling, which has practical application value.

### 5.1 The relationship between psychological fatigue and sports motivation of short track speed skaters

This study investigated the relationship between psychological fatigue and sports motivation of short track speed skaters. The results showed that psychological fatigue significantly negatively affected sports motivation, the direct effect value was −0.174, and the mediating effect was significant. The research hypothesis 1 was established.

The input model theory holds that when athletes regard their sports as a burden, they are more likely to suffer from psychological fatigue, and the burden is closely related to sports motivation. The psychological fatigue of short track speed skaters can affect the performance and cognitive ability of the body and specific motor skills (Pickering and Grgic, 2019). The results of the study based on this theory verify that the motivation of participation tendency has a significant negative predictive effect on psychological fatigue, while the motivation of avoidance tendency has a positive predictive effect on psychological fatigue (Guo, 2015). Improving the belief of short track speed skaters in unfinished tasks will stimulate stronger sports motivation, thus producing a positive promotion effect (Liu et al., 2022). On the contrary, low confidence will reduce sports motivation, indicating that psychological fatigue will negatively affect the sports motivation of short track speed skaters. Therefore, sports motivation and psychological fatigue constitute a dialectical relationship of mutual influence and mutual causality, which is in line with the core concept of the integrated model of psychological fatigue. Although previous studies have established the relationship between psychological fatigue and sports motivation, most of the conclusions are based on the analysis of athletes in different events as a whole sample, and the applicability of the conclusions in short track speed skating is not tested separately.

The theory of planned behavior believes that the more positive the individual’s attitude toward a certain behavior, the stronger the behavioral intention; the more positive the individual’s subjective norms of a certain behavior, the stronger the behavioral intention; when the attitude and subjective norms are more positive, the behavior control of individual perception is stronger; the stronger the behavior control, the stronger the behavior intention. It can be seen that the higher sports motivation of short track speed skaters, the more capable they are to avoid psychological fatigue. Therefore, the hypothesis H1 proposed in this study is based on the negative effect of psychological fatigue, and empirically proves that the psychological fatigue of short track speed skaters has a negative impact on sports motivation. On the one hand, this finding supports the conclusion that the decline of sports motivation is the concomitant phenomenon after the psychological fatigue of short track speed skaters. On the other hand, it also provides an empirical basis for the applicability of this conclusion in the group of short track speed skaters.

### 5.2 The mediating role of perceived social support

The results show that Perceived social support plays an intermediary role between psychological fatigue and sports motivation of short track speed skaters. The effect value is −0.128, and the mediating effect is significant. The research hypothesis 2 is established.

Based on the test of hypothesis H2, it is found that the psychological fatigue of short track speed skaters plays an intermediary role in the process of influencing sports motivation, and the perceived social support plays an intermediary role in the process of influencing sports motivation. It explains the 28.38% variation of the influence of psychological fatigue on the variation of sports motivation, and highlights the protective effect of perceived social support.

First of all, from the three dimensions of weakened competence in training and competition, excessive consumption of physical energy and emotional resources, and athletes’ loss of interest in sports (Raedeke, 1997), the improvement of frustration will increase the psychological distance between individuals. Irritability is easy to induce conflicts between short track speed skaters and important relationships such as teammates or coaches, while lack of sports motivation and interest in sports will make individuals resist interpersonal interaction. Researchers found that coaches and teammates are the key to the social support network of athletes in this state (Scott et al., 2024), confirmed that psychological fatigue has a negative effect on the interpersonal relationship of short track speed skaters, and confirmed the close relationship between interpersonal relationship and perceived social support (Liu and Xia, 2018), the generation of psychological fatigue will damage the interpersonal relationship of short track speed skaters, thereby reducing their subjective perception of social support.

Secondly, the sports motivation of short track speed skaters is affected by the change of perceived social support level. The change of the intensity of sports motivation usually needs to examine the two factors of individual internal demand and external environment. Perceived social support can help individuals buffer the negative effects of external stressors, reduce individuals’ awareness of the threat of stressors, reduce inappropriate behaviors, and reduce the intensity of negative emotions caused by stressors (Wang et al., 2023). The buffering hypothesis of social support holds that a high level of social support can provide individuals with important psychological resources, effectively cope with the pressure of all aspects of life, reduce the impact of negative life events, effectively supplement the lost psychological energy, and buffer the impact of negative life events on individuals. It can control its own negative emotions within a reasonable range (Li and Hu, 2023). Perceived social support can effectively meet the specific needs of short track speed skaters. Under the competitive pressure environment, meeting the relevant needs of short track speed skaters is conducive to maintaining their sports motivation level and avoiding the loss of sports motivation. Social support promotes behavioral change through a variety of mechanisms, including enhancing self-efficacy, problem-solving ability, and positive self-awareness (Sweeney et al., 2022). Improving short track speed skaters’ perception of social support is conducive to short track speed skaters’ deeper understanding of their own value, thereby enhancing the self-expectation of short track speed skaters to become outstanding in the project, and strengthening the willingness of short track speed skaters to continue to invest in sports training and competition. The findings fit the existing understanding that perceived social support can shape individual needs evaluation. In addition, the cognitive assessment theory explains the internal mechanism of the formation of intrinsic motivation. It is recommended to provide external incentives in a way that promotes the individual’s basic psychological needs, and to cultivate intrinsic motivation in terms of ability, autonomy, and social association performance (Spilsbury et al., 2023). When the basic needs of short track speed skaters in independent decision-making, competence and interpersonal attribution are met, these satisfactions will play a key intermediary role in the effect of social environmental factors on sports motivation.

Focusing on the study of short track speed skating, short track speed skaters show specific social mobility characteristics. Relevant surveys reveal that most short track speed skaters have poor family background and relatively low socioeconomic status. Cultural mobility theory believes that cultural capital is particularly important for young people from disadvantaged backgrounds; because marginalized youth are more likely to lack economic resources and social connections, cultural capital is the core means to achieve upward mobility through education (Benner et al., 2021), which makes it more likely that short track speed skaters will achieve upward mobility through sports performance. In this context, China has provided a series of policy support for high-level short track speed skaters, including education guarantee, honor incentive, retirement and resettlement, and has undertaken basic expenditures in daily life, systematic training, international competitions and other aspects. These institutional guarantees have laid a solid foundation for short track speed skaters to form a positive motivation to participate. If short track speed skaters cannot perceive such support, or lose confidence in the relevant support they obtain, it is not conducive to the formation of motivation to participate.

To sum up, short track speed skaters with strong perceived social support can effectively avoid psychological fatigue, and the negative emotions caused by psychological fatigue will not only have a negative impact on the interpersonal communication of short track speed skaters, but also make it easier for short track speed skaters to subjectively judge that they are difficult to obtain the expected social support, which makes it difficult for sports motivation to continue to invest in special training and competition. Therefore, perceived social support plays a separate mediating role between psychological fatigue and sports motivation of short track speed skaters.

### 5.3 The mediating role of sense of career calling

The results show that the sense of career calling plays an intermediary role between the psychological fatigue and sports motivation of short track speed skaters. The effect value is −0.092, and the intermediary effect is significant. The research hypothesis 3 is established.

First of all, psychological fatigue negatively affects the sense of career calling. Psychological fatigue stems from long-term high-intensity training, frequent competition pressure and other factors, resulting in slow emotional response, which in turn weakens the internal recognition of short track speed skaters on their own profession. Specifically, when short track speed skaters experience psychological fatigue, their concentration and decision-making ability decline, and they are prone to negative emotions. These negative states directly affect their sense of career calling, such as enthusiasm for sports, sense of responsibility and long-term goal pursuit. In the short track speed skating project, short track speed skaters need to face the physical challenges of high-speed skating and psychological loads such as tactical decision-making, psychological fatigue may lose interest in training and question their own value. Empirical research shows that the negative correlation between psychological fatigue and sense of career calling can be explained by self-determination theory: when the basic psychological needs cannot be met due to fatigue, the intrinsic motivation of short track speed skaters is weakened, and the sense of mission is reduced. This effect is not only reflected at the individual level, but also reduces team cohesion. Therefore, coaches should give priority to intervention in psychological fatigue, and maintain the sense of career calling through scientific training planning and psychological recovery measures to ensure the sustainable development of short track speed skaters. The higher the psychological fatigue of short track speed skaters, the lower the tendency of sense of career calling. By reducing the psychological fatigue of short track speed skaters and improving the structure and function of specific areas of the central nervous system, the sense of career calling of short track speed skaters can be effectively improved.

Secondly, the sense of career calling has become a key factor affecting the sports motivation of short track speed skaters. According to the theory of self-regulation, short track speed skaters with low sense of career calling generally have poor ability to perceived social support, and the training and competition time invested in the project is reduced accordingly, which is more likely to cause the decline of sports motivation. The sense of career calling is the deep identity of short track speed skaters to the cause of sports, which can be transformed into a strong internal driving force and stimulate positive behavior in training and competition. When short track speed skaters have a high sense of career calling, they will regard short track speed skating as the mission of life, rather than a simple career, which makes it easier for them to overcome the negative impact of psychological fatigue, they can maintain a strong sports motivation to take the initiative to seek breakthroughs even in the face of setbacks; on the contrary, a weakened sense of career calling will weaken the motivation of the movement. From a theoretical perspective, the mediating role of sense of career calling conforms to the social cognitive model, that is, as the core of the individual belief system, it regulates the influence of external pressure such as psychological fatigue on sports motivation. Under the fatigue state, the short track speed skaters with higher sense of career calling have a significantly smaller decrease in sports motivation than the short track speed skaters with lower sense of career calling. In addition, the respect of coaches can also enhance the sense of belonging of short track speed skaters, and thus enhance their sense of career calling.

### 5.4 The chain mediating role of perceived social support and sense of career calling

The results show that the total effect value of psychological fatigue on the sports motivation of short track speed skaters is −0.451, the direct effect is −0.174, the total indirect effect value is −0.277, the effect value of Ind3 is −0.057, the mediating effect is significant, and the research hypothesis 4 is established.

The mediating effect analysis showed that there was a chain mediating effect between perceived social support and professional mission in the mediating model of this study, and the relative mediating effect value was 12.64%. Although the chain mediating path of this study is significant, the effect value is much lower than the direct path. Perceived social support and sense of career calling play a chain mediating role between psychological fatigue and sports motivation, and explain the negative impact of psychological fatigue on sports motivation of short track speed skaters. The improvement of perceived social support level promotes the improvement of sense of career calling, and finally improves the sports motivation due to the improvement of sense of career calling. Social psychology believes that the social environment gives individual emotional support, which in turn guides the continuous reconstruction and improvement of beliefs, promotes the sense of career calling and makes the motivation more dynamic. The results show that perceived social support can improve the psychological fatigue of short track speed skaters and promote the positive impact on sports motivation. The perceived social support and sense of career calling included in the study are the mediating effects between psychological fatigue and sports motivation. In the process of short track speed skaters’ psychological fatigue affecting sports motivation, perceived social support and sense of career calling not only have a separate mediating effect between psychological fatigue and sports motivation, but also play a chain mediating role between psychological fatigue and sports motivation.

First of all, psychological fatigue is a continuous state of physical and mental exhaustion, which will reduce the ability of short track speed skaters to perceive the surrounding social support. In high-intensity competitive sports such as short track speed skating, long-term training and competition pressure can cause short track speed skaters to fall into the dilemma of cognitive decline, making it difficult for them to effectively identify emotional and practical support from coaches, teammates or families; individuals in the state of fatigue will give priority to the preservation of limited psychological resources, thus automatically ignoring the external support opportunities. In addition, if the psychological fatigue of short track speed skaters is not intervened in time, it will destroy the social support network and eventually amplify the negative impact of psychological fatigue.

Secondly, improving the level of perceived social support can effectively stimulate the sense of career calling, and use it as a bridge to positively regulate sports motivation and complete the chain transmission of the process of psychological fatigue. When short track speed skaters perceive sufficient social support, this external resource will be transformed into an internal driving force, strengthening their sense of identity and mission to professional roles. Specifically, strengthening the sense of career calling of short track speed skaters will directly affect sports motivation, it can not only enhance the intrinsic motivation, but also enhance the sustainability of external motivation, help short track speed skaters maintain resilience in the adversity of psychological fatigue, and fit the interaction model of social cognitive theory, that is, the supportive environment provides a positive feedback loop, encourages short track speed skaters to internalize professional goals, and then enhances their sense of career calling. In addition, the test of the chain mediating effect of this study broadens the research field of sports motivation and provides an operational framework for optimizing training interventions.

### 5.5 Limitations of the study

This study reveals the relationship between psychological fatigue and sports motivation of short track speed skaters, constructs a chain intermediary model of perceived social support and sense of career calling, and clarifies the influence of psychological fatigue on the sports motivation of short track speed skaters, which has important theoretical value for improving the sports motivation of short track speed skaters. It is suggested that during the pre-competition training period, one-to-one communication between coaches and short track speed skaters should be held once a week, focusing on information support and respect support; during the competition, strengthen peer emotional support through team review; during the recovery period after the competition, the theme sharing meeting was organized to deepen the understanding of professional mission of other short track speed skaters through the case of excellent short track speed skaters. In addition, in the process of communication, we should pay attention to the expression, feedback and intervention of psychological fatigue of short track speed skaters, so as to improve the daily psychological intervention practice of coaches.

However, due to many factors, the research still has the following deficiencies. (1) Emotion is the result of the interaction of physiological and psychological factors. The effect of psychological fatigue on the sports motivation of short track speed skaters is also diverse. Future research should further understand the influence path of psychological fatigue on the sports motivation of short track speed skaters. (2) The sample size of athletes in the three provinces may not be comprehensive enough. In addition, because the research data is derived from the subjective questionnaire test, there may be errors in the accuracy of the results. Future research should improve the research paradigm and methods, combine cross-sectional research with longitudinal research, and draw on the frontier measurement and evaluation tools of neurophysiology, brain science and other disciplines. In-depth and objective analysis of the impact of psychological fatigue on the sports motivation of short track speed skaters, while longitudinal design tracks the “psychological fatigue” dynamics of short track speed skaters. (3) Without considering more covariates, more covariates should be included in future research, such as the leadership style of coaches.

## 6 Conclusion

This study verifies the chain mediating effect of perceived social support and sense of career calling in the relationship model of psychological fatigue affecting sports motivation. Psychological fatigue can not only directly affect the sports motivation of short track speed skaters, but also indirectly affect the sports motivation through the mediating role of perceived social support and sense of career calling. The three intermediary paths are: the intermediary path of “psychological fatigue → perceived social support → sports motivation,” the intermediary path of “psychological fatigue → sense of career calling → sports motivation,” and the chain intermediary path of “psychological fatigue → perceived social support → sense of career calling → sports motivation.” Based on this study, the following conclusions are drawn: (1) There is a significant negative correlation between psychological fatigue and sports motivation of short track speed skaters. Psychological fatigue can negatively affect the sports motivation of short track speed skaters. (2) Psychological fatigue negatively affects sports motivation, perceived social support and sense of career calling; perceived social support positively affects sports motivation and sense of career calling; sense of career calling positively affects sports motivation. (3) Perceived social support and sense of career calling play a separate mediating role between the psychological fatigue and sports motivation of short track speed skaters. (4) Perceived social support and sense of career calling play a chain mediating role between psychological fatigue and sports motivation of short track speed skaters.

## Data Availability

The datasets presented in this study can be found in online repositories. The names of the repository/repositories and accession number(s) can be found in the article/supplementary material.
