# Correction: Research on the influence of psychological fatigue of short track speed skaters on sports motivation

**DOI:** 10.3389/fpsyg.2026.1814011

**Published:** 2026-03-04

**Authors:** Frontiers Production Office

**Affiliations:** Frontiers Media SA, Lausanne, Switzerland

**Keywords:** perceived social support, psychological fatigue, sense of career calling, short track speed skaters, sports motivation

The title of this article was erroneously given as: “Study on the influence of psychological fatigue on sports motivation of short track speed skaters: the chain mediating effect based on perceived social support and sense of career calling”. The correct title of the article is “Research on the influence of psychological fatigue of short track speed skaters on sports motivation”.

In the abstract, several values in the **Methods** and **Results** sections were inaccurate. This has been corrected to read:

**“Methods:** Using a variety of research methods such as questionnaire survey and mathematical statistics, 817 short track speed skaters were selected as the research objects. The “Psychological Fatigue Scale,” “Sports Motivation Scale,” “Perceived Social Support Scale,” and “Sense of Career Calling Scale” were used for psychological measurement. SPSS 27.0 and PROCESS 4.2 macro program were used to test the mediating effect, chain mediating effect and Bootstrap analysis.

**Results:** In the correlation analysis, psychological fatigue was significantly negatively correlated with perceived social support (*r* = −0.363, *p* < 0.01), sense of career calling (*r* = −0.387, *p* < 0.01), and sports motivation (*r* = −0.375, *p* < 0.01). Perceived social support was significantly positively correlated with sense of career calling (*r* = 0.494, *p* < 0.01) and sports motivation (*r* = 0.505, *p* < 0.01). There was a significant positive correlation between sense of career calling and sports motivation (*r* = 0.520, *p* < 0.01).”

A correction has been made to section **3 Research objects and methods**, subsection *3.1 Research object*, paragraph 1:

“A total of 823 questionnaires were distributed and screened after recovery. The final effective questionnaire was 817, including 611 males and 206 females. There are 465, 220, and 132 short track speed skaters aged 18–20, 21–23, 24 and above, respectively.”

A correction has been made to section **3 Research objects and methods**, subsection *3.2.1.2 Psychological fatigue scale*, paragraph 2:

“The Cronbach's α of “Psychological Fatigue Scale” in this study was 0.856, and the Cronbach's α of the three sub-dimensions was 0.912–0.931.”

A correction has been made to section **3 Research objects and methods**, subsection *3.2.1.3 Sports motivation scale*, paragraph 2:

“The Cronbach's α of the “Sports Motivation Scale” in this study was 0.812, and the Cronbach's α of the two sub-dimensions was 0.825 and 0.833, respectively.”

A correction has been made to section **3 Research objects and methods**, subsection *3.2.1.4 Perceived social support scale*, paragraph 2:

“The Cronbach's α of the “Perceived Social Support Scale” in this study was 0.892, and the Cronbach's α of the four sub-dimensions was 0.804–0.842.”

A correction has been made to section **3 Research objects and methods**, subsection *3.2.1.5 Sense of career calling scale*, paragraph 2:

“The Cronbach's α of the “Sense of Career Calling Scale” in this study was 0.879, and the Cronbach's α of the three sub-dimensions was 0.824–0.874.”

A correction has been made to section **4 Research results and analysis**, subsection *4.1 Common method bias test*, paragraph 2:

“Through the unrotational exploratory factor analysis, the results showed that there were 12 factors with eigenvalues greater than 1, and the variation explained by the first common factor was 23.629% which was less than the critical standard of less than 40% proposed by Podsakoff et al. (2003).”

A correction has been made to section **4 Research results and analysis**, subsection *4.3 Descriptive statistics and correlation analysis between variables*, paragraph 1:

“…the skewness score was −1.252 to −0.222, and the kurtosis score was −0.431 to 2.558.”

A correction has been made to section **4 Research results and analysis**, subsection *4.3 Descriptive statistics and correlation analysis between variables*, paragraph 2:

“In the correlation analysis, psychological fatigue was significantly negatively correlated with perceived social support (*r* = −0.363, *p* < 0.01), sense of career calling (*r* = −0.387, *p* < 0.01), and sports motivation (*r* = −0.375, *p* < 0.01). Perceived social support was significantly positively correlated with sense of career calling (*r* = 0.494, *p* < 0.01) and sports motivation (*r* = 0.505, *p* < 0.01). There was a significant positive correlation between sense of career calling and sports motivation (*r* = 0.520, *p* < 0.01), as shown in Table 2. The above correlation analysis results lay a good foundation for in-depth understanding of the internal relationship between variables.”

A correction has been made to section **4 Research results and analysis**, subsection *4.4 Test of chain mediating effect of perceived social support and sense of career calling*, paragraph 1:

“The PROCESS 4.2 macro program in SPSS 27.0 was used to test the mediating effect, and the demographic variables (gender and age) were controlled.”

A correction has been made to section **4 Research results and analysis**, subsection *4.4 Test of chain mediating effect of perceived social support and sense of career calling*, paragraphs 2 and 3:

“Firstly, multiple regression analysis showed that psychological fatigue had a significant negative predictive effect on sports motivation (β = −0.374, *p* < 0.001). After psychological fatigue, perceived social support and sense of career calling were included in the regression equation, the negative predictive effect of psychological fatigue on sports motivation was still significant (β = −0.144, *p* < 0.001). Psychological fatigue had a significant negative predictive effect on perceived social support (β = −0.361, *p* < 0.001), and psychological fatigue had a significant negative predictive effect on sense of career calling (β = −0.238, *p* < 0.001). Perceived social support had a significant positive predictive effect on sense of career calling (β = 0.408, *p* < 0.001), and perceived social support had a significant positive predictive effect on sports motivation (β = 0.296, *p* < 0.001). The sense of career calling had a significant positive predictive effect on sports motivation (β = 0.319, *p* < 0.001).

(…)

Perceived social support and sense of career calling play a mediating role between psychological fatigue and sports motivation, the mediating effect value is −0.277, and its 95% confidence interval is [−0.342, −0.215]. The confidence interval does not include 0, indicating that the mediating effect is significant, accounting for 61.42% of the total effect of psychological fatigue on sports motivation (−0.451). The mediating effect includes three indirect effect paths: (1) The indirect effect 1 (−0.128) produced by the way of “psychological fatigue—perceived social support—sports motivation.” and the 95% confidence interval does not contain 0, indicating that the indirect effect of the mediating variable is significant, accounting for 28.38% of the total effect of psychological fatigue on sports motivation (−0.451); (2) The indirect effect 2 (−0.092) through the path of “psychological fatigue—sense of career calling—sports motivation,” 95% of the confidence interval does not contain 0, indicating that the indirect effect of the intermediary variable is significant, accounting for 20.40% of the total effect of psychological fatigue on sports motivation (−0.451); (3) The indirect effect 3 (−0.057) through the path of “psychological fatigue—perceived social support—sense of career calling—sports motivation,” 95% of the confidence interval does not contain 0, indicating that the indirect effect of the intermediary variable is significant, accounting for 12.64% of the total effect of psychological fatigue on sports motivation (−0.451).

A correction has been made to section **5 Discussion**, paragraph 1:

“The total effect value of psychological fatigue on sports motivation was −0.451, the direct effect value was −0.174, and the total indirect effect value was −0.277.”

A correction has been made to section **5 Discussion**, subsection *5.1 The relationship between psychological fatigue and sports motivation of short track speed skaters*, paragraph 1:

“This study investigated the relationship between psychological fatigue and sports motivation of short track speed skaters. The results showed that psychological fatigue significantly negatively affected sports motivation, the direct effect value was −0.174, and the mediating effect was significant. The research hypothesis 1 was established.”

A correction has been made to section **5 Discussion**, subsection *5.2 The mediating role of perceived social support*, paragraphs 1 and 2:

“The effect value is −0.128, and the mediating effect is significant. The research hypothesis 2 is established.

Based on the test of hypothesis H2, it is found that the psychological fatigue of short track speed skaters plays an intermediary role in the process of influencing sports motivation, and the perceived social support plays an intermediary role in the process of influencing sports motivation. It explains the 28.38% variation of the influence of psychological fatigue on the variation of sports motivation, and highlights the protective effect of perceived social support.”

A correction has been made to section **5 Discussion**, subsection *5.3 The mediating role of sense of career calling*, paragraph 1:

“The effect value is −0.092, and the intermediary effect is significant.”

A correction has been made to section **5 Discussion**, subsection *5.4 The chain mediating role of perceived social support and sense of career calling*, paragraphs 1 and 2:

“The results show that the total effect value of psychological fatigue on the sports motivation of short track speed skaters is −0.451, the direct effect is −0.174, the total indirect effect value is −0.277, the effect value of Ind3 is −0.057, the mediating effect is significant, and the research hypothesis 4 is established.

The mediating effect analysis showed that there was a chain mediating effect between perceived social support and professional mission in the mediating model of this study, and the relative mediating effect value was 12.64%.

There was a mistake in Figure 2 as published. Figure 2 has now been removed.

There was a mistake in Table 1 as published. There was a discrepancy in the number of decimal places. The corrected Table 1 appears below.

**Table 1:** Gender differences between different variables.

**Variant**	**Gender**	**Quantity**	***M***	**SD**	***F***	***P***
Psychological fatigue	Male	611	3.23852	0.681801	2.174	0.155
	Female	206	3.31521	0.631577		
Perceived social support	Male	611	4.98670	0.810157	0.652	0.923
	Female	206	4.98058	0.727667		
Sense of career calling	Male	611	3.82443	0.626203	0.272	0.512
	Female	206	3.79126	0.634077		
Sports motivation	Male	611	2.84561	0.819938	0.496	0.439
	Female	206	2.79531	0.763166		

There was a mistake in Table 2 as published. There was a discrepancy in the number of decimal places. The corrected Table 2 appears below.

**Table 2:** Descriptive statistics and correlation analysis between variables.

**Variables**	***M***	**SD**	**Psychological fatigue**	**Perceived social support**	**Sense of career calling**	**Sports motivation**
Psychological fatigue	3.25785	0.669942	1			
Perceived social support	4.98516	0.789739	−0.363^**^	1		
Sense of career calling	3.81607	0.627973	−0.387^**^	0.494^**^	1	
Sports motivation	2.83293	0.805837	−0.375^**^	0.505^**^	0.520^**^	

There was a mistake in Table 3 as published. There was a discrepancy in the number of decimal places. The corrected Table 3 appears below.

**Table 3:** The regression analysis of perceived social support and sense of career calling between Psychological Fatigue and sports motivation.

**The regression equation**		**Overall fitting index**			**The significance of regression coefficient**		
**Outcome variables**	**Predictive variables**	***R***	*R* ^2^	***F***	β	**SE**	***t***
Sports motivation	Gender	0.375	0.140	44.267^***^	−0.008	0.060	−0.261
	Age				−0.003	0.035	−0.089
	Psychological fatigue				−0.374	0.039	−11.481^***^
Perceived social support	Gender	0.366	0.134	41.870^***^	0.015	0.059	0.463
	Age				−0.047	0.034	−1.429
	Psychological fatigue				−0.361	0.039	−11.050^***^
Sense of career calling	Gender	0.542	0.294	84.615^***^	−0.010	0.043	−0.327
	Age				−0.006	0.025	−0.189
	Psychological fatigue				−0.238	0.030	−7.525^***^
	Perceived social support				0.408	0.025	12.866^***^
Sports motivation	Gender	0.608	0.369	94.933^***^	−0.012	0.052	−0.424
	Age				0.019	0.030	0.672
	Psychological fatigue				−0.144	0.037	−4.651^***^
	Perceived social support				0.296	0.034	8.992^***^
	Sense of career calling				0.319	0.043	9.610^***^

^*^p < 0.05, ^**^p < 0.01, ^***^p < 0.001.

There was a mistake in Table 4 as published. There was a discrepancy in the number of decimal places. The corrected Table 4 appears below.

**Table 4:** Analysis of the mediating effect of perceived social support and sense of career calling in the influence of psychological fatigue on sports motivation.

**Effect situation**	**Effect value**	**Boot standard error**	**Boot CI lower limit**	**Boot CI upper limit**	**Relative mediating effect**
Gross effect	−0.451	0.039	−0.527	−0.374	
Direct effect	−0.174	0.037	−0.247	−0.101	38.58%
Total indirect effect	−0.277	0.032	−0.342	−0.215	61.42%
Indirect effect 1(Ind1): psychological fatigue-perceived social support-sports motivation	−0.128	0.021	−0.173	−0.090	28.38%
Indirect effect 2(Ind2): psychological fatigue-sense of career calling-sports motivation	−0.092	0.016	−0.125	−0.063	20.40%
Indirect effect 3(Ind3): psychological fatigue-perceived social support -sense of career calling-sports motivation	−0.057	0.012	−0.082	−0.036	12.64%

The original version of this article has been updated.

